# Deformation Prediction of Unstable Slopes Based on Real-Time Monitoring and DeepAR Model

**DOI:** 10.3390/s21010014

**Published:** 2020-12-22

**Authors:** Mei Dong, Hongyu Wu, Hui Hu, Rafig Azzam, Liang Zhang, Zengrong Zheng, Xiaonan Gong

**Affiliations:** 1 College of Civil Engineering and Architecture, Zhejiang University, Hangzhou 310058, China; 11512022@zju.edu.cn (H.W.); gongxn@zju.edu.cn (X.G.); 2 Hangzhou Ruhr Technology Co., Ltd., Hangzhou 310023, China; huhui@ruhrtec.cn (H.H.); zhengzr@ruhrtec.cn (Z.Z.); 3 Sino-German Resources Environment and Geo-Hazards Research Center, North China University of Water Resources and Electric Power, Zhengzhou 450056, China; azzam@lih.rwth-aachen.de; 4 Department of Engineering Geology and Hydrogeology, RWTH-Aachen University, 52074 Aachen, Germany; zhangl1228@126.com

**Keywords:** landslides, monitoring system, deformation prediction, early warning, DeepAR model

## Abstract

With increased urbanization, accidents related to slope instability are frequently encountered in construction sites. The deformation and failure mechanism of a landslide is a complex dynamic process, which seriously threatens people’s lives and property. Currently, prediction and early warning of a landslide can be effectively performed by using Internet of Things (IoT) technology to monitor the landslide deformation in real time and an artificial intelligence algorithm to predict the deformation trend. However, if a slope failure occurs during the construction period, the builders and decision-makers find it challenging to effectively apply IoT technology to monitor the emergency and assist in proposing treatment measures. Moreover, for projects during operation (e.g., a motorway in a mountainous area), no recognized artificial intelligence algorithm exists that can forecast the deformation of steep slopes using the huge data obtained from monitoring devices. In this context, this paper introduces a real-time wireless monitoring system with multiple sensors for retrieving high-frequency overall data that can describe the deformation feature of steep slopes. The system was installed in the Qili connecting line of a motorway in Zhejiang Province, China, to provide a technical support for the design and implementation of safety solutions for the steep slopes. Most of the devices were retained to monitor the slopes even after construction. The machine learning Probabilistic Forecasting with Autoregressive Recurrent Networks (DeepAR) model based on time series and probabilistic forecasting was introduced into the project to predict the slope displacement. The predictive accuracy of the DeepAR model was verified by the mean absolute error, the root mean square error and the goodness of fit. This study demonstrates that the presented monitoring system and the introduced predictive model had good safety control ability during construction and good prediction accuracy during operation. The proposed approach will be helpful to assess the safety of excavated slopes before constructing new infrastructures.

## 1. Introduction

Numerous slope failures have been recorded with respect to human activities, making slope stability analysis a continued and interesting topic in the field of geotechnical engineering [1,2]. In China, a large number of unstable steep slopes have been formed due to the rapid development of high-speed motorways and railways in mountainous terrain. Landslides and rockfalls pose a risk to traffic and cause high losses every year. In order to overcome the serious and complex challenges of geohazards induced by construction work, monitoring techniques and early-warning methods have become of interest in dealing with slope failure [3,4,5,6].

Currently, the assessment of landslide behavior is usually undertaken by means of monitoring. Robotic Total Stations (RTS) are broadly used by the scientific community for the monitoring of unstable slopes [7,8]. Satellite- and ground-based technologies, such as Synthetic Aperture Radar (SAR) and Synthetic Aperture Radar Interferometry (InSAR), can be used to identify the slopes in movement with high accuracy [8,9,10,11,12]. Airborne Light Detection and Ranging (LiDAR), combined with interference elimination data preprocessing, is mainly used in landslide investigation to provide high-resolution point clouds of the topography, find ancient landslides, detect mass movements and monitor landslides, as well as to assess hazards [13,14]. In recent years, the Global Navigation Satellite System (GNSS) has become fully operational and can broadly monitor the surface displacement of slopes [15,16,17,18]. Distributed Optical Fiber Sensors (DOFS) are currently widely used for the monitoring of landslides [19]. A wireless monitoring system with Microelectromechanical Sensors (MEMS) can be used to automatically measure the gravity-induced joints, displacement and environmental factors of a slope [20]. Images of a slope can be obtained by high-resolution cameras periodically and then the entire slope stability and displacement of feature points can be analyzed by image recognition technology [21,22,23,24,25,26]. Internet of Things (IoT) technology is a good way to address the conditions of the entire unstable area during the emergency response stage of a moving slope without causing risks to humans.

Slope deformation is an external manifestation of a combination of multiple factors, such as land type, geological condition, rainfall, fluctuation in groundwater level, extent of vegetation and seismic loads. At present, research on the prediction and early warning of landslide disasters is mainly divided into regional landslide susceptibility prediction [27,28] and single landslide deformation prediction [29,30,31]. Regional landslide susceptibility prediction focuses on the spatial probability of landslide occurrence in a given region [32]. Single slope displacement prediction models can be divided into physical concept models [33,34] and data-driven models [35]. For single landslides with complex terrain conditions and geological structures, it is often difficult to obtain exact physical and mechanical parameters for rock, soil and water information. The predicted results of physical models are sometimes not reliable enough to derive the deformation laws of landslides [36]. Thus, data-driven models, which analyze only the input and output variables to predict the development of landslide displacement, are sometimes more applicable [37]. Data-driven models can be further divided into the following five categories: probability analysis models, heuristic models, deterministic models, mathematical statistical models and artificial intelligence models [38,39]. With the development of system science and nonlinear science, nonlinear characteristics of the time series of slope displacement have been deeply studied. Therefore, artificial intelligence models are becoming increasingly popular lately. With comprehensive consideration of the landslide displacement and external inducing factors, artificial intelligence models with many input variables are able to predict the future evolution of a landslide. Artificial intelligence models use Support Vector Machines (SVM) [40], Artificial Neural Networks (ANN), fuzzy logic and decision trees to perform nonlinear fitting and to find a nonlinear relationship between environmental factors and landslide susceptibility. For example, Akgun et al. (2012) [41] used fuzzy logic theory to predict landslides. Bui et al. (2016) [42] used SVM, ANN and kernel logistic regression models to predict shallow landslides and to compare the pros and cons of the prediction results. Li et al. (2010) [43] used a Back Propagation Neural Network (BPNN) to predict the displacement of Baishuihe landslide under the influence of rainfall and reservoir water level. Huang et al. (2016, 2017) [44,45] analyzed the factors influencing the landslide deformation of a reservoir bank and used the extreme learning machine model to predict the landslide displacement of the reservoir bank. Hong et al. (2015) [46] used a binary classification kernel logic model, decision tree model and SVM model to predict landslides in Yihuang, China. Cai et al. (2015) [47] optimized the least squares support vector machine for landslide displacement prediction by using a genetic algorithm that considered multiple influencing factors. Dong et al. (2018) [31] used a time series analysis combined with a 3D geological model and a finite-element simulation method to improve the reliability of the calculated factor of safety. The acquisition of environmental factors, spatial analysis and data management of deformation prediction of unstable slopes were mainly based on monitoring technology. Despite these studies, the application of artificial intelligence models for deformation prediction is still a new multidisciplinary research field.

This study proposes a comprehensive monitoring system based on the IoT and other devices, such as GPS benchmarks, inclinometers, tilt sensors, crack meters, etc., to control the safety of the excavated unstable slope in the emergency stage. It introduces an artificial intelligence algorithm based on time series and probabilistic forecasting for the prediction of deformation based on real-time displacement and rainfall data. The applicability and accuracy of the monitoring system and prediction model are verified against the field data from an excavated slope along the Qili connecting line of the Hangchang high-speed motorway in Quzhou City, Zhejiang Province, China. A monitoring system collected field data in the slopes from December 2015. The field data obtained from the monitoring system and prediction of deformation by the proposed model have greatly assist in the construction of the Qili connecting line and in early warnings for landslides during the operation period.

## 2. Study Area

### 2.1. Location and Geological Setting

**Figure 1:**
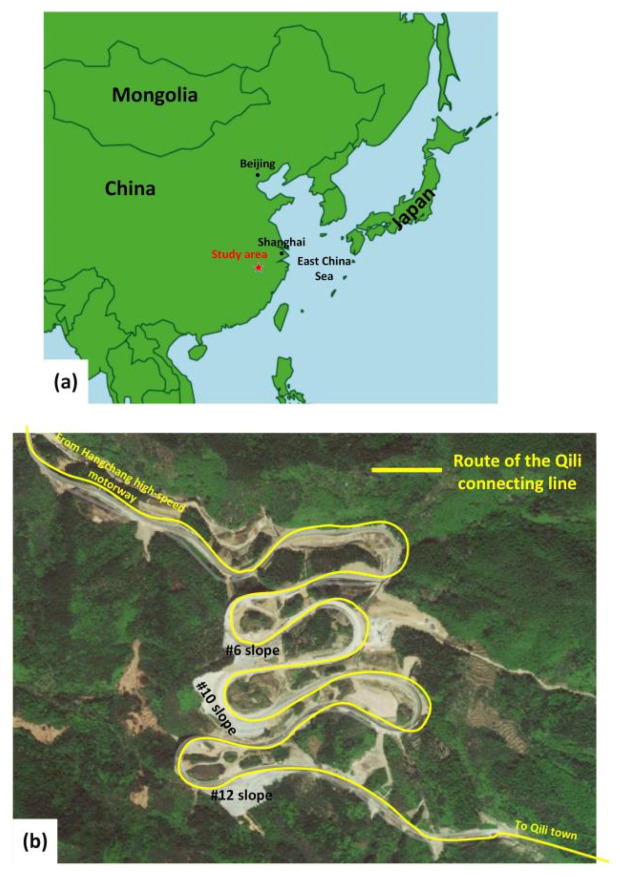
Location of the study area (**a**) and the route of the construction project (**b**).

The study area was located in the Yangtze River Basin on the southeastern coast of China, 430 km southwest of Shanghai International Airport (Figure 1a). The construction project connects the Hangchang high-speed motorway to Qili town in Quzhou City in Zhejiang Province, China (yellow curve in Figure 1b). The total length of the route is 7.725 km. During the construction process, three high-steep slopes (#6, #10 and #12 in Figure 1b) were formed. The terrain where the route is located is dominated by high-steep mountains, with the highest point at 1050 m.a.s.l. and the base at 437 m.a.s.l. Excluding limited Quaternary deposit, the study area is composed of Upper Jurassic breccia, tuff, conglomerate, sandstone and mudstone. The Lower Ordovician calcareous mudstones, carbonaceous mudstones and marls are beneath the Upper Jurassic rocks. The lithology of the project area from top to bottom includes gravel with clay, strong weathered glutenite and mudstone, moderately weathered sandstone and glutenite, strong weathered mudstone, moderately weathered mudstone, carbonaceous mudstone, sandstone and glutenite. The region is located between the Shangfang–Yeling syncline and the Qianligang syncline. It is a northwest-inclined monoclinic formation. The site is mainly affected by the Jiehu–Xiushu fault. Faults, joints and cracks are strongly developed along the project site and thus the integrity of the rock masses is very poor.

### 2.2. Climate Setting

**Figure 2:**
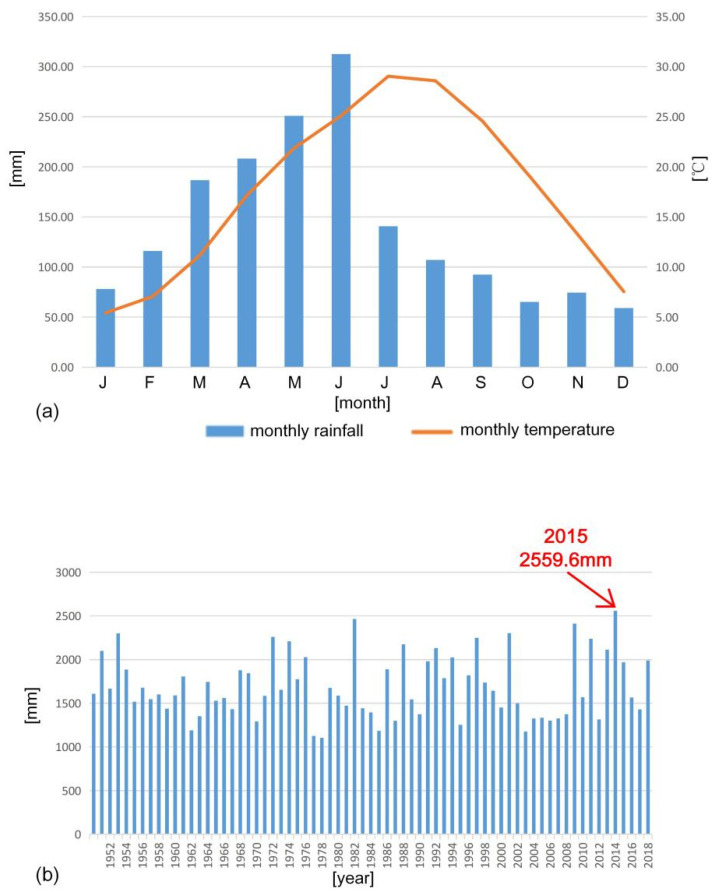
Rainfall and temperature distribution in Quzhou City: (**a**) Climatograph constructed by averaging monthly rainfall data recorded since 1951 and temperature data available since 1951; (**b**) Historic record of annual rainfall in Quzhou City from 1951 to 2019.

Quzhou City (8844.79 km^2^), in which the study area is located, belongs to the subtropical monsoon climate zone, warm and humid. According to the recorded climate data from 1951 to 2019 (Figure 2), the average annual temperature is 17.5 °C and the monthly temperature averages range from 5.43 °C (January) to 29.04 °C (July). The region receives on average 1690.89 mm of precipitation annually. In the year 2015, which is the year the project was implemented, the accumulative precipitation reached 2559.6 mm in Quzhou City. The monthly rainfall levels can be seen in Table 1. In the project site, the annual average precipitation is 1804.92 mm. The wet season is from February to July, especially concentrated between May and July. From the end of July to the following January is the dry season. Typhoons and heavy rains happen occasionally between August and October.

### 2.3. Field Survey

**Figure 3:**
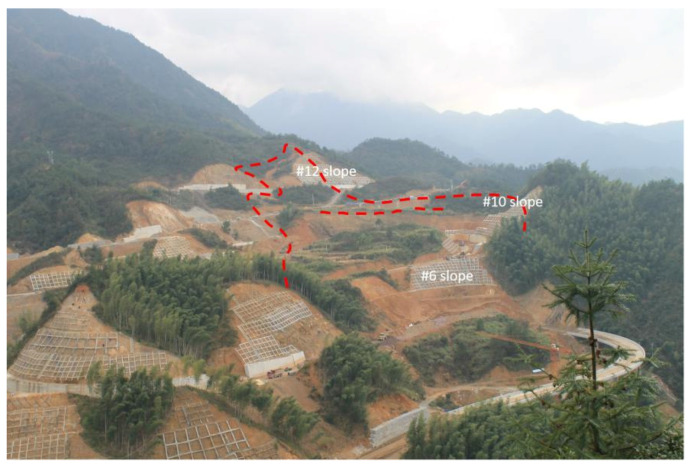
Overview of the project site.

The excavation of slopes #6, #10 and #12 started in August 2014. During the excavation period from August 2014 to November 2015, several cracks were investigated surrounding the #6, #10 and #12 high-steep slopes around the route (red dotted line in Figure 3). The #6 slope was made of strong weathered sandstone and mudstone covered by gravel with diluvial clay. A subgrade passed over the top of the #6 slope. The #10 slope was made of Jurassic rock masses. The filling subgrade passed over the top of the #10 slope and the maximum filling height was about 7 m. The #12 slope was made of Jurassic rock masses. Beneath were the Ordovician mudstone, carbonaceous mudstone and sandstone. The toe of the #12 slope was strongly weathered. In November 2015, the investigated individual gravity-induced joints connected together to become a large and continuous crack. The study area suffered from rainfall washout frequently during the construction and operation periods (see the monthly rainfall levels in Table 1).

**Figure 4:**
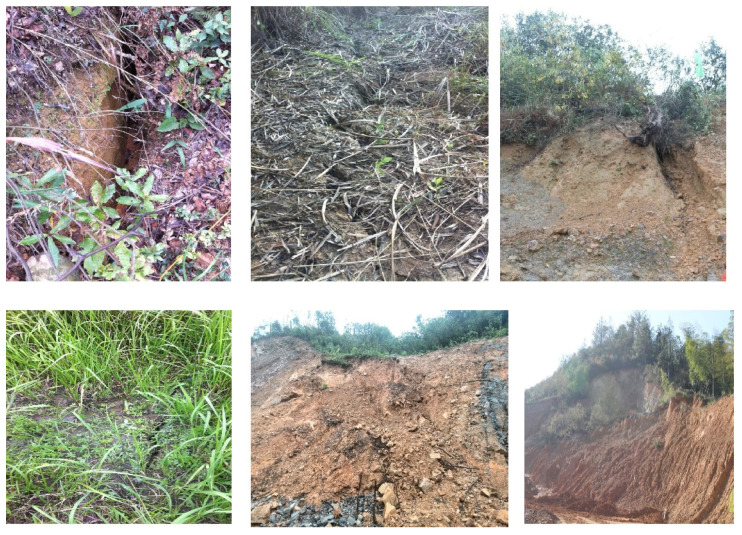
Cracks and deformation in the field.

**Figure 5:**
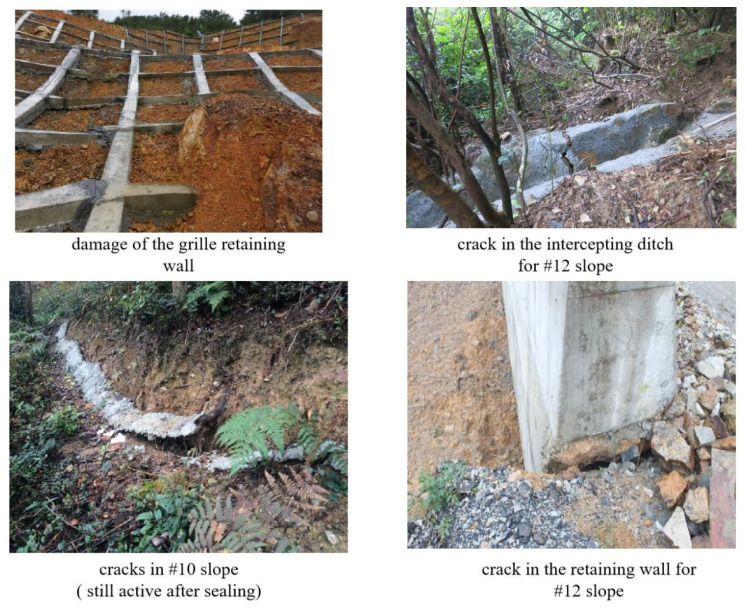
Cracks and deformation after disposal measurement.

In-situ photos of some cracks are shown in Figure 4. Although engineering treatments, such as surface drainage, underground drainage, load reduction and back pressure, were performed, the cracks were expanding and interconnecting until December 2015 (shown in Figure 5). An on-site inspection and preliminary survey results in December 2015 revealed that cracks surrounding the #6, #10 and #12 slopes were linked. Rock masses encountered many dislocations and the mountain showed a trend of potential overall slope failure.

Cracks in the #6, #10 and #12 slopes were attributed to the combined action of unloading at the slope foot, loading at the slope top, change of groundwater level and disturbance in the geological structure during the construction process. Unloading was mainly caused by the excavation of the slope. The higher the excavation, the more extensive the breaking of the rock masses and the greater the impact were. Loading was mainly caused by the filling of road subgrade. Since the Qili line had many bends along the slope, filling of the subgrade in the upper slope affected the stability of the lower slope. After the excavation, water was released and the groundwater level decreased, which was beneficial to the stability of the slope. However, lowering of the water level also caused consolidation and settlement of the shallow loose soil. In December 2015, a comprehensive real-time slope stability monitoring network was established on the site to provide important technical support for the design of the next treatment plan and on-site construction safety during the emergency period.

## 3. Monitoring System and Acquired Data

### 3.1. Monitoring System

**Figure 6:**
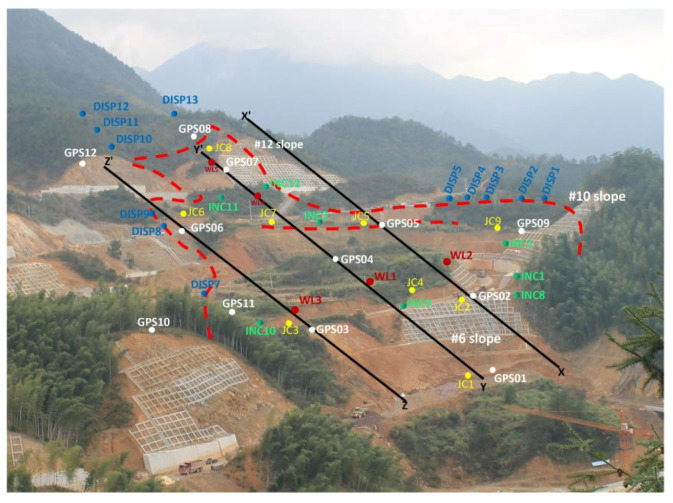
Deployment of the monitoring system in the project site, in which the red dashed dots indicate the landslide outline. White circles indicate the GPS benchmarks, yellow circles indicate the deep inclinometers, green circles indicate the tilt meters, dark blue circles indicate the crack meters and dark red circles indicate the groundwater level indicators.

The deployment of monitoring devices is shown in Figure 6. It was designed based on the results of the field survey and the existing geological survey. Due to the large volume and wide range of potential sliding bodies on the project site, both the overall and local deformation characteristics were considered. Three monitoring profiles were investigated, covering the entire landslide body (*X*-*X*′, *Y*-*Y*′, *Z*-*Z*′ in Figure 6). The monitored parameters included displacement in the surface, displacement in the depths, width of gravity-induced joints, meteorological data and groundwater level.

(1) Surface displacement measurement

The surface displacements of the landslides were determined using long-term GPS observations. Data were acquired every two hours from December 2015 up to the time of writing. A GPS network with thirteen benchmarks was installed, including one reference point, of which eight benchmarks (GPS 02, 03, 04, 05, 06, 07, 09 and 11 in Figure 6) were installed inside of the sliding block, four benchmarks were positioned outside of the crack circle (GPS 01, 08, 10 and 12 in Figure 6) and one reference station (cannot be seen in Figure 6) was positioned in the stable area far from the sliding block. GPS 08 was installed outside of the unstable blocks on the upper edge of the #12 slope to monitor the displacement trend of the whole project site. Real Time Kinematic (RTK) GPS receivers capable of tracking the L_1_C/A, L_1_C, L_2_C, L_2_E, L_5_ code on 220 channels were used to detect displacements over the sliding area. The RTK technique was employed to acquire the three-dimensional coordinates, deformation orientation of superficial displacement, amount of deformation and deformation velocity of monitoring points. The data accuracy was improved by using a differential algorithm and the data was transmitted to the data center through a Data Transfer Unit (DTU). By using a differential algorithm, the daily RTK accuracy could reach 1 mm + 1 ppm in the horizontal direction and 2 mm + 1 ppm in the vertical direction. The distances among the reference station and monitoring points were less than 1 km.

(2) Surface tilt measurement

Tilt sensors were installed on the retaining walls and other stress concentration positions. MEMS with high sensitivity were adopted. The change of slope angle was collected by a two-axis tilt sensor. The dynamic response was captured using the acceleration sensor. The monitoring accuracy could reach 0.1° and 0.001 g, respectively. Monitoring frequency was once per minute, which is high-frequency dynamic acquisition.

(3) Deep displacement measurement

From December 2015 to September 2016, nine deep displacement monitoring holes were drilled. Manual incline measurement and a fixed inclinometer were adopted. Deep inclinometers were embedded to indicate the internal displacement of the slope, as well as to determine the potential sliding surface of landslides. The installation depth of the inclinometer pipes reached the stable bedrock. The accuracy of the fixed inclinometer was 0.1° and the monitoring frequency was adjustable. In Figure 6, JC1, 3, 4, 6, 7 and 9 used manual incline measurement. Fixed inclinometers were installed at the positions of JC 2, 5 and 8. The installed depth is shown in Table 2.

(4) Crack deformation measurement

The string displacement meter was adopted to monitor the discovered cracks in the surface. The crack’s width was measured by displacement or crack meter in real time. The deformation value and the deformation rate could be obtained. The monitoring accuracy was 0.1~0.2 mm. The monitoring frequency was once per hour.

(5) Groundwater level monitoring

In the project site, five water level indicators (red solid circles in Figure 6) were installed in the drilled observation holes. They were powered by solar energy and were fitted with smart data acquisition devices to receive and send signals collected by sensors. Storage management and data analysis were performed on the system platform.

Thus, an extensive monitoring system was established to acquire data on the research site. The monitoring system started to work in the slopes from December 2015 and continues at the time of writing.

### 3.2. Acquired Data

#### 3.2.1. GPS Data

In 2016, the 3D displacement of GPS 01 was about 8~10 mm, indicating that the high-steep slope on the Qili connection line was unstable. The planar displacements of GPS 02 and GPS 03 in the #6 slope were 13.47 mm and 17.58 mm, respectively. The displacement changes of GPS 03 mainly occurred during the early monitoring period (January 2016).

**Figure 7:**
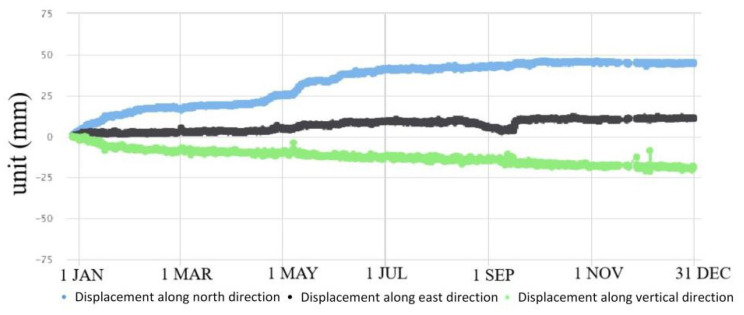
Displacements recorded by GPS 04 in 2016.

**Figure 8:**
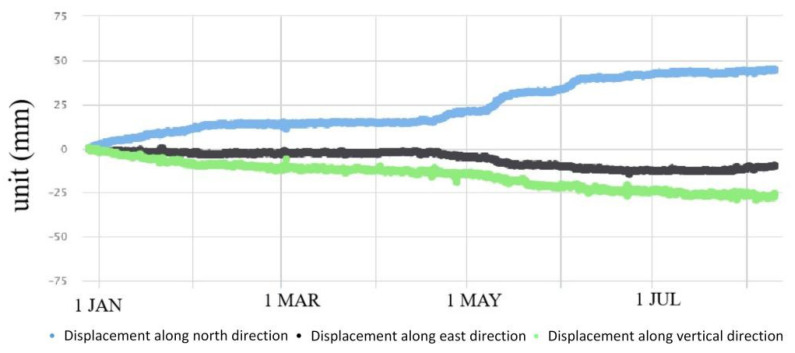
Displacements recorded by GPS 05 in 2016.

**Figure 9:**
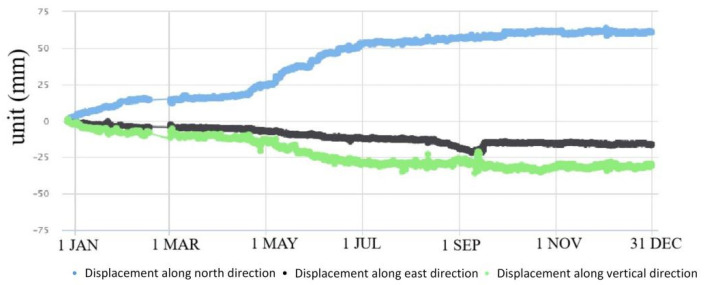
Displacements recorded by GPS 06 in 2016.

**Figure 10:**
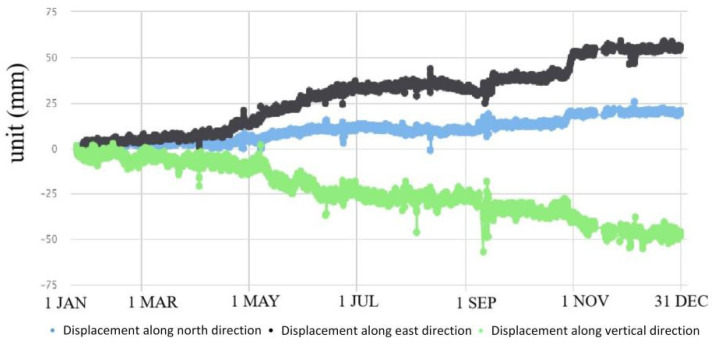
Displacements recorded by GPS 09 in 2016.

GPS 04, 05, 06 and 09 had significant displacement trends, accompanied by surface subsidence (Figure 7, Figure 8, Figure 9 and Figure 10). GPS 04 recorded 14 mm displacements to the north in the first month of monitoring. From 31 January to 20 April, the deformation rate reduced and the total displacement to the north was 7 mm during this time. From 21 April to 7 June the deformation rate increased due to heavy rainfall of 637 mm. Then the deformation rate turned stable. GPS 05 recorded 13 mm displacements to the north in the first month of monitoring. From 4 February to 20 April the deformation rate was stable. From 21 April to 7 June the deformation rate increased due to heavy rainfall and the total displacement to the north was 24 mm during this time. GPS 05 was removed due to construction work in August. GPS 06 moved 13.6 mm to the north in the first month of monitoring. From 31 January to 20 April, the deformation rate was stable. The displacement to the north reached 41 mm during the rainy season from 21 April to the end of June. The slope where GPS 09 was installed showed significant deformation and subsidence. The device moved toward north by 20 mm, east by 60 mm and in a vertical direction by 50 mm.

GPS 07 showed significant changes in position during the monitoring period and the displacement to the north reached 71 mm in April 2017, when the equipment was removed. The recorded 3D displacements of GPS 08, 10, 11 and 12 were less than 10 mm, which also indicated that the entire slope was relatively not seriously unstable. Based on the real-time monitoring data, necessary engineering disposal measures, such as slope unloading, subgrade raising, retaining walls, a square bolt frame and an anchor cable lattice, were undertaken during the construction period. Thus, the deformation of the slopes was mitigated during 2017.

#### 3.2.2. Crack Meter Data

Data of the width propagation of the monitored cracks are shown in Table 3.

From Table 3 we can see that the DISP 06 installed on the east retaining wall of the #6 slope showed no obvious development. The displacement of other crack meters increased considerably (about 20–50 mm) and the development trend was relatively consistent. In January 2016 the displacement was affected by snow melting. In April, May and June it was affected by heavy rainfall and then the development rate increased. In the other months the deformation rate was stable and grew slowly. DISP 10, 11, 12 and 13 were removed on 27 April as the construction work was urgently performed due to the significant deformation measured.

#### 3.2.3. Tilt Sensor Data

The data from tilt sensors were stable and there was no abnormal change in the inclination angle. The cumulative inclination angle of X and Y axes was within ±1°. The inclination sensors were all located on the landslide masses, which indicated that the local deformation of each slope did not connect to an entire landslide.

#### 3.2.4. Deep Inclinometer Data

The deep inclinometer data and the manual measured data showed that the JC 4 and JC 8 were stable. The deformation in JC 1 occurred at depths of 21 m, 23 m and 25 m; in JC 3 at depths of 15.5 m, 19.5 m, 23.5 m, 26 m and 29 m; in JC 6 at a depth of 7.5 m; in JC 7 at a depth of 22.5 m; in JC 9 at a depth of 15 m. The monitored data indicated that the #10 and #12 slopes had significant sliding trends.

### 3.3. Analysis of the Triggered Factors

In order to find the significant triggering factors of the deformation, the monitored data of the study area was analyzed. The 3D deformations of GPS 04, 05, 06 and 07 reached 5 mm, 5 mm, 5.7 mm and 5.9 mm, respectively, from 19 to 25 April 2016. The average velocity was 0.83~0.98 mm/d. The widths of crack meters DISP 01–05 and 10–13 also showed significant increases from 20 to 21 April, ranging from 0.56 mm to 7.89 mm. The average daily rainfall in April was 11.55 mm. On 20 April the daily rainfall reached 55.5mm. The groundwater level in WL 2, 3 and 5 increased by 1–1.5 m.

The 3D displacements of GPS 04, 05 and 06 reached 6.8 mm, 8.3 mm and 9.3 mm, respectively, from 6 to 13 May 2016. The average velocity was 0.97~1.33 mm/d. The vertical displacement of GPS 09 was 10 mm. The widths of crack meters DISP 01–05 reached 4.15 mm, 7.55 mm, 1.02 mm, 1.74 mm and 1.43 mm, respectively, from 11:00 pm of 6 May 2016, to 9:00 am of 7 May 2016. On 6 May the daily rainfall reached 53 mm. The heavy rain lasted from 6 to 10 May, while the deformation data faded after one week.

The widths of crack meters DISP 01–05 and 08 reached 5.18 mm, 8.88 mm, 0.83 mm, 3.8 mm, 1.38 mm and 2.41 mm, respectively, from 3:00 to 12:00 am of 29 May. At 3:00 am the rainfall reached 30.5 mm/h (56 mm over the whole day). The widths of crack meters DISP 01–05 and 08 reached 2.48 mm, 3.75 mm, 0.46 mm, 1.16 mm, 0.94 mm and 1.76 mm, respectively, from 3:00 to 12:00 am of 29 May. At 8:00 am the rainfall reached 41 mm/h (67 mm over the whole day).

The 3D displacement of GPS 14 reached 11.1 mm from 6:00 am of 30 June to 10:00 am of 1 July. The study area received 104 mm rainfall from 28 to 29 June.

**Figure 11:**
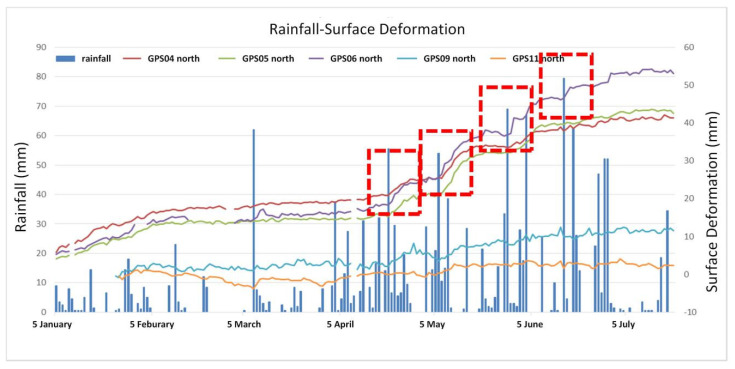
Record of rainfall and surface deformation in the #10 slope.

**Figure 12:**
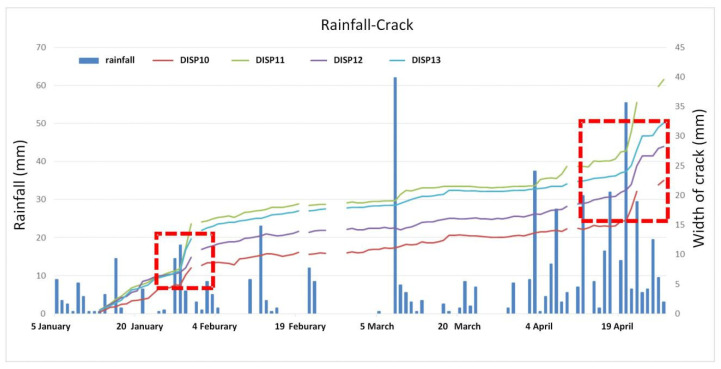
Record of rainfall and crack width in the #12 slope.

The above analyses demonstrate that the monitored significant deformation of the study area was influenced by heavy rainfall. The data from monitored devices installed in the #10 and #12 slopes in the rainy season of 2016 are compared with the rainfall in Figure 11 and Figure 12.

Looking at all the monitored data, it can be seen that the deformation of the #6 slope was not significantly influenced by rainfall. Rainfall can be determined as the main triggering factor of the surface deformation and crack raising of the #10 and #12 slopes (Figure 11 and Figure 12).

## 4. Method of Deformation Prediction

The construction work of the Qili connection line was completed in May 2018 and the line became operational in October 2018. Twelve GPS stations, eight crack meters, four groundwater level indicators and six deep inclinometers were retained on the site to collect data from the #6, #10 and #12 slopes. The surface displacement was increased mainly due to rainy weather. Especially from the beginning of April to the end of July 2019, heavy and long-duration rainfall was concentrated on the area and the soil became saturated (Table 1). Consequently, the rate of surface and deep displacement increased significantly. Thus, the precipitation data was chosen as an input variable to predict the deformation. From 2016 to 2019, the recorded data from GPS benchmarks were continuous. Thus, the surface displacement was chosen as the output data in the current study. In order to establish an early warning system for the danger of possible slope failure on the motorway, a Probabilistic Forecasting with Autoregressive Recurrent Networks (DeepAR) [38] model was adopted in the current study.

DeepAR is a time series prediction method based on deep learning proposed by Amazon in 2017 [48]. It is a methodology for producing accurate probabilistic forecasts based on the training of an autoregressive recurrent network model on a large number of related time series. It can effectively learn a global model from relevant time series and can learn complex patterns, such as seasonality and uncertainty growth of data over time, so as to predict the time series.

During the training stage, the input of each time of the model includes the real value of the previous time and the feature X of the current time. X could be a feature of the current time. After learning the neural network, the hit returned by the network is functionally affine and the prediction probability distribution of the current time is returned.

In the prediction stage, the input of each time includes two parts: the prediction value of the previous time and the covariate of the current time, which is a recursive prediction method. Then, the learned network is used to predict the time series.

Three evaluation indicators are normally used to reflect the predictive effect of an algorithm, namely the Mean Absolute Error MAE, the Root Mean Square Error (RMSE) and the goodness of fit. The MAE is used to evaluate the degree of closeness between the predicted value and the actual monitored value. The smaller the value of the MAE, the better the fit. The RMSE calculates the square root of the mean of the sum of squares of the sample points corresponding to the predicted value and the actual monitored value. The goodness of fit (*R*^2^) is also called the coefficient of determination and reflects the degree of fit of the predicted value to the actual monitored value. Its value range of 0–1. A value of 0 means no fit and a value of 1 means a perfect fit.

The training and testing data set are shown in Table 4. The training dataset includes 16,000 data in total. Data from GPS benchmarks and crack meters show that landslide deformation was closely related to precipitation in the rainy season. The main characteristic from 2016 to 2019 was the creep deformation.

## 5. Results and Discussion

**Figure 13:**
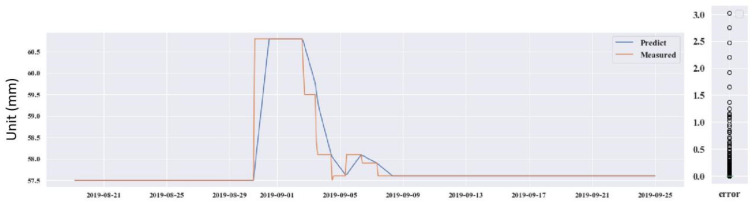
Predicted and measured planar displacement of GPS 04.

**Figure 14:**
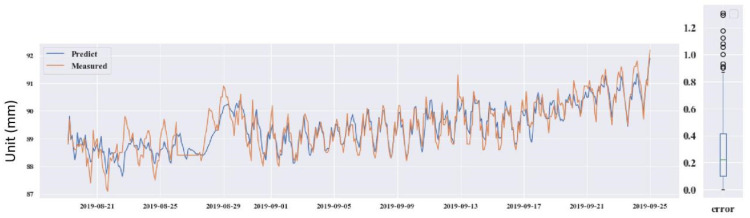
Predicted and measured planar displacement of GPS 06.

**Figure 15:**
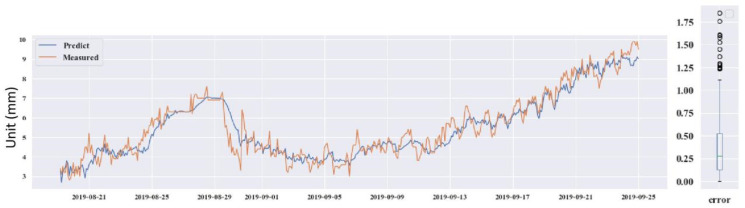
Predicted and measured planar displacement of GPS 12.

**Figure 16:**
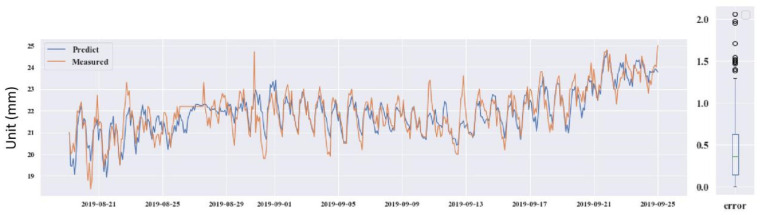
Predicted and measured planar displacement of GPS 14.

The four testing GPS devices were numbered as GPS 04, 06, 12 and 14. GPS 04, 06 and 12 were retained after the construction period. GPS 14 was installed on 21 June 2016, after the protection construction work finished, in the #12 slope. Looking through the data from the operation of the monitoring system to now, it can be seen that the deformations on the four GPS stations were induced by the rainfall. Thus, precipitation was chosen as the input influence index, which was also analyzed by Wu et al. (2018) [49]. The prediction used the DeepAR model. The predicted results for the four GPS devices are shown in Figure 13, Figure 14, Figure 15 and Figure 16.

The prediction results were evaluated by the three indicators MAE, RSME and R^2^. Table 5 shows the evaluated results.

Through predicting the deformation of GPS 04, 06, 12 and 14, the predictive ability of the DeepAR model in the instability slopes has been demonstrated. The MAE and RMSE values are small and the R^2^ values are close to 1. However, some measured and predicted data cannot be matched (see in Figure 13, Figure 14, Figure 15 and Figure 16). The local abnormal prediction might be caused by a dependent variable, external disturbance of the construction work or temperature excursion of a pressure sensor.

During the excavation from August 2014 to August 2015, several cracks were investigated surrounding the #6, #10 and #12 high-steep slopes of the Qili connecting line. The deformation continued even after the treatment was undertaken. Thus, in December 2015, a comprehensive monitoring system considering local and entire deformations to acquire high-frequency data was proposed and installed in the project site. The monitoring program was adjusted according to the construction work. The monitored devices worked together to address the changes of the slopes and gave real-time suggestions for the protection disposals.

GPS benchmarks were installed to monitor the surface displacement of the entire sliding area and individual slopes. GPS 01 was installed at the bottom of the slope, which was an important position to know the overall stability of the slope. The monitoring data showed that the overall state of the slope was unstable and the accumulated 3D displacement was 8–10 mm. The local deformation of the project site was obvious, especially at the #10 and #12 slopes. The 3D displacement of GPS 09 installed on the #10 slope reached 102 mm by the end of 2017. The 3D displacement of GPS 07 installed on the #12 slope reached 109 mm towards the northwest. The monitoring data of the other GPS benchmarks installed on the slopes reflected the similar characteristics of local deformation. In addition to the observation of the overall and local deformation, the monitoring data also reflected the movement characteristics of the landslide in different time periods. For example, from the end of March to the end of June 2016, the precipitation increased significantly. The GPS benchmarks (04, 05, 06, 07 and 09) installed inside the sliding mass showed different degrees of accelerated deformation characteristics. The deformation rate notably exceeded 1 mm/day at the #10 (GPS 09) and #12 (GPS 07) slopes. At the end of August 2016, the antisliding piles and anchor rod of the #12 slope were finished. Therefore, during the subsequent continuous rainfall season, the data from GPS 07 did not show accelerated deformation. GPS 09 at the #10 slope showed accelerated deformation in the following several continuous rainfall periods.

Crack meters were installed to master the width propagation of pre-existing cracks. The monitoring data of cracks showed obvious seasonal characteristics. The crack width increased especially from March to June. In the dry season, cracks did not expand very much. The deformation characteristics were related to the rainfall distribution and consistent with GPS monitoring data.

The change of slope angle was collected by two-axis tilt sensors. The data from the tilt sensors were stable, which indicates that the local deformation of each slope did not connect to an entire landslide.

Manual incline measurement and fixed inclinometers were adopted to monitor the internal displacement of the slopes, as well as to determine the potential sliding surface of landslides. Data from JC1, JC3, JC6, JC 7 and JC9 all revealed the position of the deep sliding surface, which can support the position of antisliding piles.

The assessment based on the long-term monitoring data showed that the high-steep slopes of Qili connecting line had the typical landslide characteristics. The boundary of the landslide mass and the main deformation position in the deep part were detected. The activity of the front of the slope was weak and that of the back was strong. Data from GPS benchmarks and crack meters showed that landslide deformation was closely related to precipitation in the rainy season (see the analysis in Section 3.3). The slip zone was partially connected in the middle and back part of the landslide. From the long-term monitoring data, it can be seen that the main characteristic was the creep deformation. According to the assessment from the monitoring data, the antisliding treatment measures were implemented step by step for the landslide from June 2016.

Since the monitoring system installed for the project of the Qili connecting line had abundant measured data, long time span, good detection frequency and rich variables (including rainfall, water level, temperature, displacement, cracks and dip angle), it was a good case to be selected as an attempt at a data-driven model for the prediction of deformation. During the construction period, the deformation was induced by the rainfall, the natural condition of the slope and the excavation. In the operation period of the Qili connecting line, since 2018, creep deformation was dominant. Prediction of the deformation was more important to avoid serious disasters. The DeepAR model was applied as a tool for forecasting the creep landslide in the current study. However, the generalization ability of the DeepAR model for the landslide needs to be optimized with more time and monitoring data. In the study area, a managing platform which can receive, store and display real-time monitoring data from the sensors has been established. The deformation prediction module with DeepAR model will be embedded in the managing platform. The predictive results could support decision-making with regard to necessary engineering disposals or warning people.

## 6. Conclusions

In this paper, an IoT-based monitoring system, combining multiple sensors such as GPS benchmarks, crack meters, tilt sensors, deep inclinometers and water level indicators, was successfully implemented during construction and operation of the Qili connecting line of an important motorway in the mountainous area of Zhejiang Province, China. The monitoring system considered the entire deformation and the local deformation of the high slopes. During construction, data from the monitoring system assisted in the in-situ protection disposals of the instability slopes. During the operation period, a DeepAR-based data-driven model was adopted to predict displacement early. The prediction of unstable slopes was based on the data collected by the advanced monitoring technology. The proposed method is an effective approach to perform comprehensive monitoring and to establish an early-warning system for the safety of infrastructure involving steep slopes.

## Figures and Tables

**Table 1:** Monthly rainfall levels in the study area from 2014 to 2019 (unit: mm).

Year	M	F	M	A	M	J	J	A	S	O	N	D
2014	26.1	140.3	216.7	124.3	315.8	553.1	203.8	313.5	58.0	14.6	133.2	14.3
2015	55.0	155.7	125.7	152.0	389.7	530.7	362.4	151.2	98.4	165.0	246.1	127.7
2016	111.3	45.3	111.3	357.6	349.7	478.8	27.1	34.3	191.1	130.9	95.7	34.4
2017	34.1	48.3	202.9	218.7	137.4	572.3	41.1	104.1	15.0	14.0	127.3	51.6
2018	93.1	47.5	187.3	252.6	150.8	181.8	51.2	113.8	58.5	47.0	118.6	128.7
2019	124.6	276.4	234.7	180.3	180.7	314.7	468.6	87.4	9.5	28.4	8.6	76.6

**Table 2:** Installed depth of the fixed inclinometers.

Inclinometer	Installed Depth (m)
JC 2	3.5, 17, 18.5, 20.5, 22.5, 30.5
JC 5	6, 18, 20, 22, 41, 43
JC 8	9.5, 14, 16.5, 22.5, 23.5, 27, 30.5

**Table 3:** Record of the crack propagation.

Crack Meter	Width Propagation (mm)
DISP 01	41.03
DISP 02	62.99
DISP 03	35.61
DISP 04	53.99
DISP 05	63.26
DISP 06	0
DISP 07	9.56
DISP 08	21.44
DISP 09	21.2
DISP 10	22.76
DISP 11	39.79
DISP 12	28.57
DISP 13	32.42

**Table 4:** Training and testing dataset.

Equipment Type	Number of Testing Devices	Frequency of Data Acquisition	Training Dataset	Testing Dataset
GPS benchmarks	4	Every 2 h or 1 h	Since 1 January 2016 to 20 August 2019, 16,000 data in total	Since 21 August 2019 to 25 September 2019, 510 data in total

**Table 5:** Evaluation of the predictive ability of the DeepAR model for four GPS devices.

Devices	MAE (mm)	RMSE (mm)	R^2^
GPS04	0.0931384	0.346316	0.860973
GPS06	0.286219	0.37134	0.823638
GPS12	0.371732	0.494544	0.911876
GPS14	0.427168	0.559492	0.735154

## Data Availability

The data presented in this study are available on request from the corresponding author. The data are not publicly available due to privacy restrictions.
